# The modern Russian pharmaceutical market: consumer attitudes towards distance retailing of medicines

**DOI:** 10.1186/s12913-022-07991-7

**Published:** 2022-04-30

**Authors:** Liudmila Lobuteva, Alisa Lobuteva, Oksana Zakharova, Oxana Kartashova, Natalia Kocheva

**Affiliations:** Department of Organization and Economics of Pharmacy, I.M. Sechenov First Moscow State Medical University (Sechenov University), Moscow, Russian Federation

**Keywords:** Consumer behaviour, Distance selling of medicines, Internet pharmacy, Pharmaceutical product

## Abstract

**Background:**

In Russia, remote retail trade of over-the-counter (OTC) medicines was legalised. According to statistics as of April 2020, consumer demand in the categories of “online pharmacies” increased by 803%.

**Methods:**

The study was conducted in two stages by cross-sectional method using a structured questionnaire in the central region of Russia: 1st stage - July-August 2020; 2nd stage - February-March 2021. The results of the study were obtained using qualitative (method of discussions in focus groups) and quantitative methods (survey) of sociological research, logical and statistical analysis. The representativeness of the data was ensured by a sufficient sample size including 1194 consumers (with confidence probability = 0.95 and confidence interval ≤ 0.05).

**Results:**

The number of respondents fully supporting the legalisation of online trade in medicines increased. Consumer attitudes towards online commerce depend on the age group of the respondents. 1.5 times more respondents over 46 years (12.1%) are strongly against distance selling of medicines compared to survey participants aged 18 to 25 years (7.8%). Six months after the first survey, no respondent strongly opposed the sale of prescription medicines through the internet, whereas in the first survey half of consumers held this view. The percentage of respondents who considered pharmaceutical counselling when purchasing medicines online as extremely important decreased by a factor of 4 over time (10.9%) M (08.2020) = 3.66 (0.992); M (03. 2021) = 3.17 (0.981) t = 7.66 (*p* < 0.05). Consumers consider accessibility for people with disabilities (80.3%) to be the most significant advantage of distance selling medicines.

**Conclusion:**

Consumer demand for the purchase of medicines online will grow as this type of sale has undeniable advantages. However, some risks remain when buying medicines online.

## Background

One of the most relevant areas in the Russian pharmaceutical market today is the remote retail sale of medicines. The sale of medicines online has a rich history, especially in the USA, where Internet pharmacies appeared in early 1990s. Since then, many countries have developed their own Internet pharmacies and online medicine sales. Most distance selling is done by ordering the required medicine online and then delivering it.

In the European Union (EU) medicines can be purchased online. Consumers should only buy medicines from Internet pharmacies registered with the national competent authorities in EU Member States to reduce the risk of purchasing substandard or fake medicinal products. To this end, the European Commission designed a dedicated logo that appears on the websites of registered Internet pharmacies. By clicking on the logo, the purchaser will be redirected to the page of that pharmacy in the list of legally operating online pharmacies and retailers [1].

Germany represents the largest online pharmacy market, accounting for approximately 56% of the market share in 2019. The UK and France are, respectively, the second and third largest remote retail medicine markets [2].

Scandinavian countries are also actively developing the online pharmacy market. For example, the total e-commerce market in Sweden is estimated to be at least EUR 9 billion as of 2021. Pharmaceutical e-commerce is estimated to be around EUR 500–600 million, which represents around 6% of the total e-commerce market [3].

In the UK, the number of online pharmacies increased almost by 7 times between 2009 and 2020 (from 56 to 390) [4].

China is considered one of the most advanced countries that has been actively developing distance selling of medicines for several decades. As of 2019, it is estimated that online sales of medicines in China amounted to RMB 13.8 billion, 115 times more than in 2012 (RMB 0.12 billion) [5].

In Russia, according to the legislation, the distance selling of medicines includes the receipt, formation, storage and delivery of orders for medicines, as well as their dispensing.

In the government of the Russian Federation, remote retailing of medicines has been actively discussed since 2017. Legislators tried to build a new system for selling and delivering medicines to consumers. At that time a draft law on online sale of pharmaceuticals passed the first stage of approval. But then its consideration was suspended.

Consideration of the draft law resumed in 2020, in part due to pressure from circumstances surrounding the COVID-19 pandemic. Parliament Members considered the government draft law on online sales of medicines at the second stage of approval on 31 of March. On April 3 the Federal Law of April 3, 2020 No. 105-FZ [6] was passed, amending the Federal Law of April 12, 2010 No. 61-FZ “On the Circulation of Medicines” [7] and Article 15.1 of Federal Law No. 149-FZ of July 27, 2006 “On Information, Information Technology and Information Protection” [8].

On 18 of May, the Government of the Russian Federation published Resolution No. 697 of May 16, 2020 “On Approval of the Rules for Issuing Permission to Conduct Retail Trade in Medicinal Products for Medical Use by Distance Selling, Conducting Such Trade and Delivery of the Medicines to Citizens and Amending Certain Acts of the Government of the Russian Federation Regarding Retail Trade in Medicinal Products for Medical Use by Distance Selling” [9].

In accordance with this Resolution, only pharmacies holding a pharmaceutical license and having obtained a special permit can carry out distance sales of medicines. The permit itself is issued by the Federal Service for Healthcare Supervision (Roszdravnadzor). The rules also detail requirements for the content of the website of a pharmacy organisation selling pharmaceuticals remotely. Medicines can be delivered to the purchaser’s place of residence, stay or actual location or another address indicated by the purchaser. Medicines may be delivered by employees of the pharmacy or by third-party couriers engaged by the pharmacy on the basis of an agreement. The third-party courier company must have special equipment for delivery of medicines, including for maintaining a required temperature. The rules do not contain any requirements for qualifications of couriers, their pharmaceutical or other special education [9–13].

Currently, only non-prescription medicines are allowed by default in the Russian Federation for distance selling. Prescription medicines can be sold remotely in emergency circumstances if the government decides so. Narcotic, psychotropic medicines and medicines containing more than 25% of alcohol cannot be sold remotely under any circumstances.

Following the adoption of relevant regulations, statistics show that 12% of Russians ordered from online pharmacies for the first time in April 2020, with an 830% increase in consumer enquiries in the “online pharmacy” categories [14].

Thus, a current importance and relevance of the topic has determined the purpose of this study - to identify consumer attitudes towards the remote retail sale of medicines on the Russian pharmaceutical market.

## Methods

The purpose of this study was to identify the features of remote medicines retail trade in the Russian pharmaceutical market.

The objects of the study were 1194 questionnaires filled out by consumers. The authors used the method of cross-sections on specially developed structured questionnaires, the method of logical analysis, qualitative (method of discussion in focus groups) and quantitative methods (survey, questioning) of sociological research. Focus group discussion was undertaken to test the questionnaire and identify shortcomings. The discussion was attended by 3 groups of 6 respondents disposed by age. Participants in the study filled out a questionnaire in advance. The discussion went on for an hour. The transcription of the data collected was followed by a comparative analysis of the respondents’ quotes. By using the focus group method, a “pilot study” was carried out, which helped to test the respondents’ understanding of the interpretation of the concepts and the meaning of the questions before collecting the data, and helped to confirm the relevance of the research topic.

For this study, a continuous sample was used, covering all units of the general population. Respondents filled out the questionnaires on their own. Two sociological surveys were conducted as part of the study: the first in July–August 2020 (792 questionnaires) and the second in February–March 2021 (402 questionnaires).

The survey of consumers was carried out using a specially designed questionnaire consisting of two modules. The first stage in the development of the questionnaire was the analysis of the topic of the survey and the formulation of questions, then a pilot questionnaire was developed with a predominance of open questions. At the next stage of the development of the questionnaire, a pilot survey and analysis of its results were carried out. And the final stage of developing the questionnaire was to clarify the wording of the instructions and the content of the questions. In total, the questionnaire included 2 modules.

The first module of the questionnaire includes questions describing the composition of respondents by age.

The second module of the questionnaire consists of three groups of questions. The first group of questions seeks to identify respondents’ views on the legalisation of distance selling of over-the-counter, prescription, narcotic and psychotropic medicines.

The second group of questions aims to explore the frequency with which consumers purchase medicines online, as well as what consumers perceive as changes in medicine prices in online segment of the market.

The third group of the questions contains multidirectional questions. The respondents’ answers to these questions reveal consumers’ opinions about the importance of pharmaceutical counselling in the selection of medicines in online pharmacies and the details of delivery of medicines to customers, as well as the strengths and weaknesses of online sales, as perceived by consumers.

All questions in the questionnaire are in closed or semi-closed form. Closed questions allow respondents to choose from ready-made answers (multiple or single choice) or answers on a point scale. Semi-closed-ended questions allow respondents to choose from ready-made answers with the option of responding independently.

To determine the reliability of the results obtained in this study, the method is based on the definition of the so-called Student’s criterion t (reliability coefficient). Its value is determined by the ratio of the difference between the compared average values to the error of their difference. The error of the difference is equal to the square root of the sum of the squares of the average errors of the compared values.

The results of the processing of respondents’ opinions provided detailed data on consumer attitudes towards remote retailing of pharmaceuticals.

The results of the study were processed and systematised using qualitative and quantitative methods of sociological research and descriptive statistics, using Microsoft Office Excel 2019 programs.

The representativeness of data at 95% confidence level and ± 5% margin of error was ensured by a random, simple sample of a large size (1194 respondents), as well as by the use of a modern statistical package.

## Results

The results obtained by applying the method of discussion in focus groups are reflected in Table 1.

**Table 1:** Identification of the strengths of distance retailing through focus group discussions

Anticipated strengths of distance retailing of medicines	Respondent’s opinion (%)
Accessibility for the disabled	100
Accessibility for the elderly	89
Possibility to order medicines at any time	83.33
Possibility to order medicines to anywhere	83.33
Opportunity to order medicines without interrupting work	55.55
Opportunity to order medicines while doing personal business	66.66
Sefety during pandemic	94.44
Ability to check the availability of medicines when ordering	55.55
Ability to order different products in one place (on one website)	61.11
Possibility of self-selection of medicines from a wide range	66.66
Lack of pharmacy worker influence on consumer choice	72.22
Lack of promotional items	27.77
Saving money	44.44
Possibility to buy medicines from home in bad weather	61.11
Ability to obtain a large amount of information about the medicines	61.11
Confidence in the effectiveness and safety of medicines	50
No strengths	16.66

Thus, during the discussion, it was determined that “accessibility for the disabled” and “accessibility for the elderly” should be combined into “accessibility for people with disabilities”, “the ability to order medicines at any time” and “the ability to order medicines to anywhere” is also combined into one answer: “the ability to order a drug at any time and place”. Respondents suggested that the strengths of “the ability to order drugs without interrupting work” and “the ability to order drugs while doing personal business” can be combined into “saving time”. “The lack of promotional items” was not approved by the respondents as a strong point of the distance selling of medicines. It was also proposed to replace “the ability to make a purchase of medicines from home in bad weather” with “The ability to make a purchase of medicines in comfortable conditions” (Table 2).

**Table 2:** Identification of the weaknesses of distance retailing by the method of discussion in focus groups

Anticipated weaknesses of distance retailing of medicines	Respondent’s opinion (%)
The risk of receiving an ineffective medicine	61.11
The risk of receiving an unsafe medicine (expired)	61.11
Lack of confidence in the eligibility of organization which trading medicines remotely	44.44
Increased risk of drug abuse	38.88
Increased risk of self-treatment	44,.44
Possible lack of delivery to hard-to-reach places	50.00
Difficulties in self-selection of medicines due to the presence of analogues	33.33
The possibility of buying unnecessary medicines and wasting money	50.00
Lack of consultation with a pharmacist when delivering medicines	61.11
Possible overpricing of medicines	33.33
Impossibility of ordering narcotic and psychotropic drugs	33.33
Impossibility of ordering poisonous and potent drugs	27.77
The process of making an order and buying medicines is slowing down	33.33
Technical problems in the work of Internet	38.88
Lack of social contacts when buying medicines online	16.66

Based on the results obtained, only 16.6% of respondents consider the lack of social contacts during the purchase of medicines online as a weakness of the distance selling of medicines, therefore this anticipated weakness was not included in the survey. The discussion participants also suggested that it would be appropriate to combine “the risk of receiving an ineffective medicine” and “the risk of receiving an unsafe medicine” into “the risk of receiving an ineffective and unsafe medicine”, as well as “increased risk of drug abuse” and “increased risk of self-treatment” in “increased risk of drug abuse and harm to health.” “Impossibility to order narcotic and psychotropic drugs” and “Impossibility to order poisonous and potent drugs” were also combined.

The results of processing the responses to the first module of the questionnaire allowed to create a profile of the respondents who participated in the study.

A total of 1194 consumers took part in the survey: 792 respondents in July–August 2020 and 402 respondents in February–March 2021.

The findings indicate that in the two surveys (08.2020 and 03.2021), the number of consumers with a positive attitude towards remote retailing of medicines increases: M (08.2020) = 3.82 (0.966); M (03.2021) = 4.02 (0.897); t = 3.377 *p* < 0.05.

Changes in the attitudes of different age groups of consumers towards remote retailing of pharmaceuticals over the analysed period are visualised in Table 1.

Table 3 shows that among consumers aged 18 to 25 there is an almost twofold increase in the number of those who believe they are more likely to support online sales of medicines. At the same time, the number of respondents who are indifferent to innovations on the pharmaceutical market almost halves.

**Table 3:** Evolution of consumer opinions (%) in different age groups towards online sales of medicines for the period 08.2020 to 03.2021

Consumer attitudes	18–25 years old		26–45 years old		46 or more years old	
	08.2020	03.2021	08.2020	03.2021	08.2020	03.2021
Fully supportive.	38.3	35.7	32.9	27.8	12.1	14.3
Rather, I support	25.2	42.9	22.8	38.9	24.2	42.9
Neutral	19.1	10.1	24.3	22.2	18.2	7.1
Rather, I do not support	9.6	11.3	14.3	11.1	33.4	35.7
Categorically against	7.8	–	5.7	–	12.1	–

In the group of consumers aged 26 to 45 there is a more than 1.5-fold increase in the number of respondents who are rather supportive of the reform. At the same time, the number of respondents who are rather not in favour of online sales of pharmaceuticals is decreasing.

The number of respondents in the age group of consumers over 46 has almost doubled, who are more likely to support the innovations in the medicines trade. At the same time the percentage of indifferent consumers has more than halved.

It should be emphasised that in all age groups of consumers, not a single respondent to the second survey in February–March 2021 spoke out strongly against the sale of medicines via the internet. This differs significantly from the attitudes expressed by consumers participating in the first survey in July–August 2020.

Thus, in all three age groups of consumers, there is a trend towards positive attitudes towards distance retailing of medicines.

The present study allows us to trace changes in consumer opinion regarding the legalisation of distance retailing for three categories of medicines (medicines that are dispensed from a pharmacy without a doctor’s prescription, with a doctor’s prescription, and those containing narcotic medicines and psychotropic substances). The results of the study are visualised in Table 4.

**Table 4:** Evolution of consumer perceptions (%) of online trade in the three product categories over the period from 08.2020 to 03.2021

Feature	Over-the-counter medicines		Prescription medicines		Narcotic medicines and Psychotropic substances	
	08.2020	03.2021	08.2020	03.2021	08.2020	03.2021
Fully supportive.	31.7	43.5	9.2	19.6	4.6	2.2
Rather, I support	45.9	34.7	28.0	32.5	7.3	10.9
Neutral	5.5	19.6	5.0	37.0	4.6	17.4
Rather, I do not support	1.8	2.2	6.9	10.9	4.6	26.0
Categorically against	15.1	–	50.9	–	78.9	43.5

It was found that over the period under analysis there was an almost 1.5-fold increase in the number of consumers who fully support remote retail sales of over-the-counter medicines. The number of respondents with a neutral attitude to such reforms in the OTC trade increased by more than 3.5 times.

Over the analysed period the number of consumers who fully support the legalisation of distance selling of prescription medicines has doubled. The number of consumers who are indifferent to the sale of prescription medicines via the internet has increased by more than 7 times. At the same time, the number of consumers who are rather not in favour of distance selling of prescription medicines has increased by more than 1.5 times.

There was a significant change in the opinion of consumers who were categorically against the distance selling of OTC and prescription medicines. None of the respondents in February–March 2021 spoke out strongly against the legalisation of online sales of prescription and over-the-counter medicines. However, in the July–August 2020 survey, half of the consumers were categorical. However, in the July–August 2020 survey, half of consumers were strongly against the remote sale of prescription medicines and almost one in six consumers was against the sale of over-the-counter medicines.

The number of respondents who were categorically against the possible legalisation of remote retail sales of medicines containing narcotic medicines and psychotropic substances decreased by more than 1.5 times. At the same time, the number of hesitant consumers, who indicated that they would rather not support the online sale of such groups of medicines, increased almost sixfold. The number of respondents who were indifferent to such innovations increased significantly.

It is thus established that, between July–August 2020 and February–March 2021, the number of consumers with a negative attitude towards the legalisation of distance selling of prescription, over-the-counter, narcotic and psychotropic medicines has decreased, while the number of consumers with a positive or neutral attitude towards this reform has increased.

**Fig. 1:**
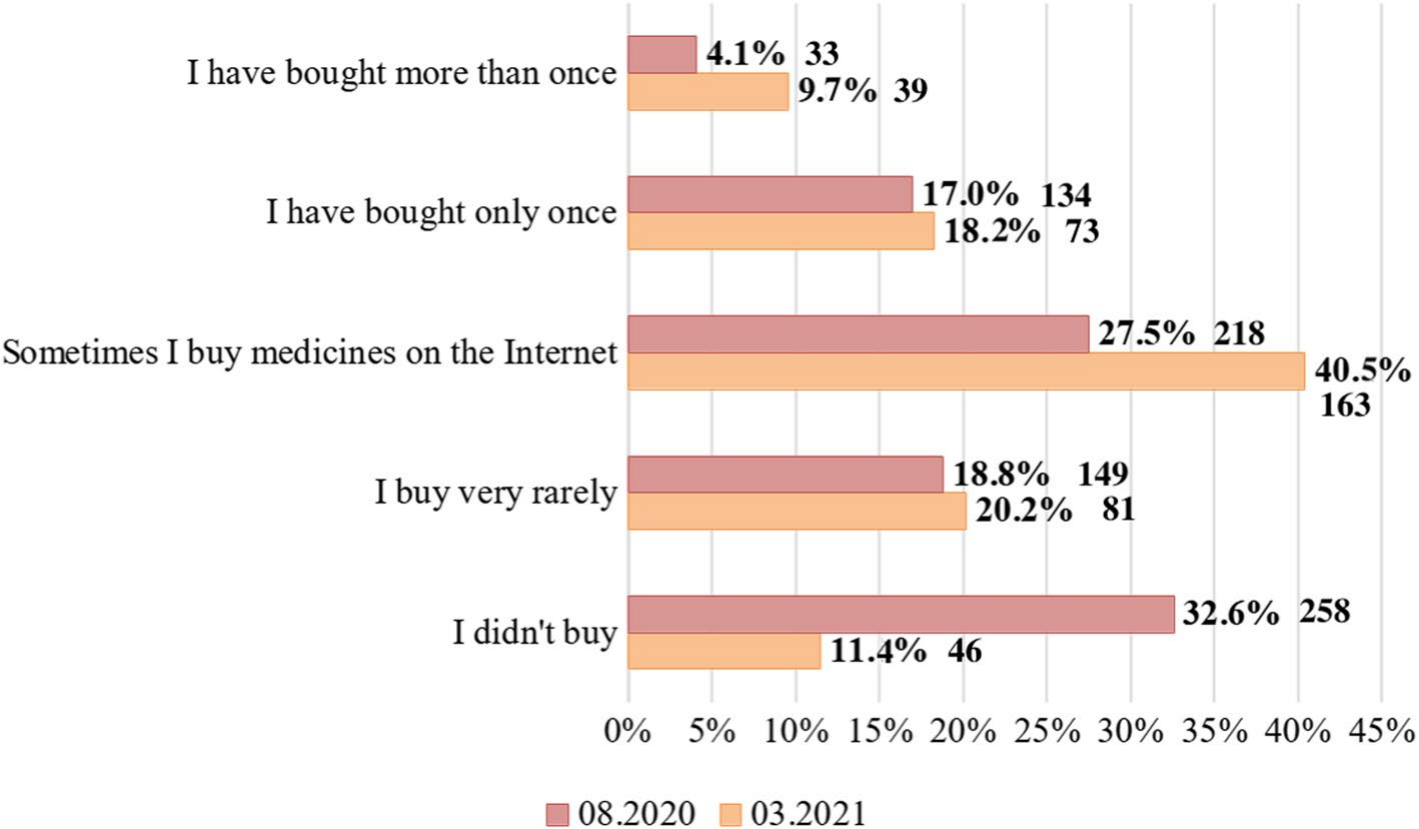
Evolution of consumer demand (%) for remote purchase of medicines from 08.2020 to 03.2021

This study has identified significant changes in consumer demand for distance purchasing of medicines (Fig. 1).

It was found that between July–August 2020 and February–March 2021, the number of respondents who had never bought any medicines remotely decreased by almost 3 times. At the same time, the number of consumers who have repeatedly purchased medicines online has more than doubled, and the number of consumers who have sometimes purchased medicines remotely has increased by 1.5 times.

**Fig. 2:**
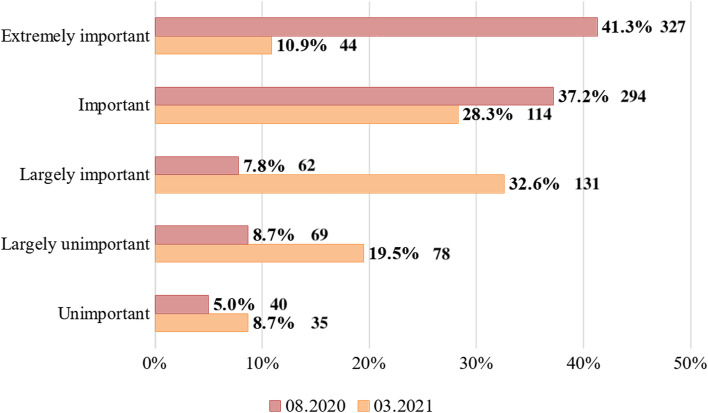
Evolution of consumer opinion (%) from 08.2020 to 03.2021 on the relevance for them of pharmaceutical counselling when purchasing medicines online

Figure 2 shows how important the information received from the pharmacy worker (pharmacist) is for consumers when choosing a medicine over the Internet.

It was found that over the period under analysis the possibility of pharmaceutical counselling remains important for consumers when purchasing medicines online, but the degree of this importance is changing. Thus, the number of consumers who consider the possibility of pharmaceutical counselling to be largely important for them has increased by more than 4 times. At the same time, the number of consumers who still consider such an opportunity to be extremely important has decreased by almost 4 times.

**Fig. 3:**
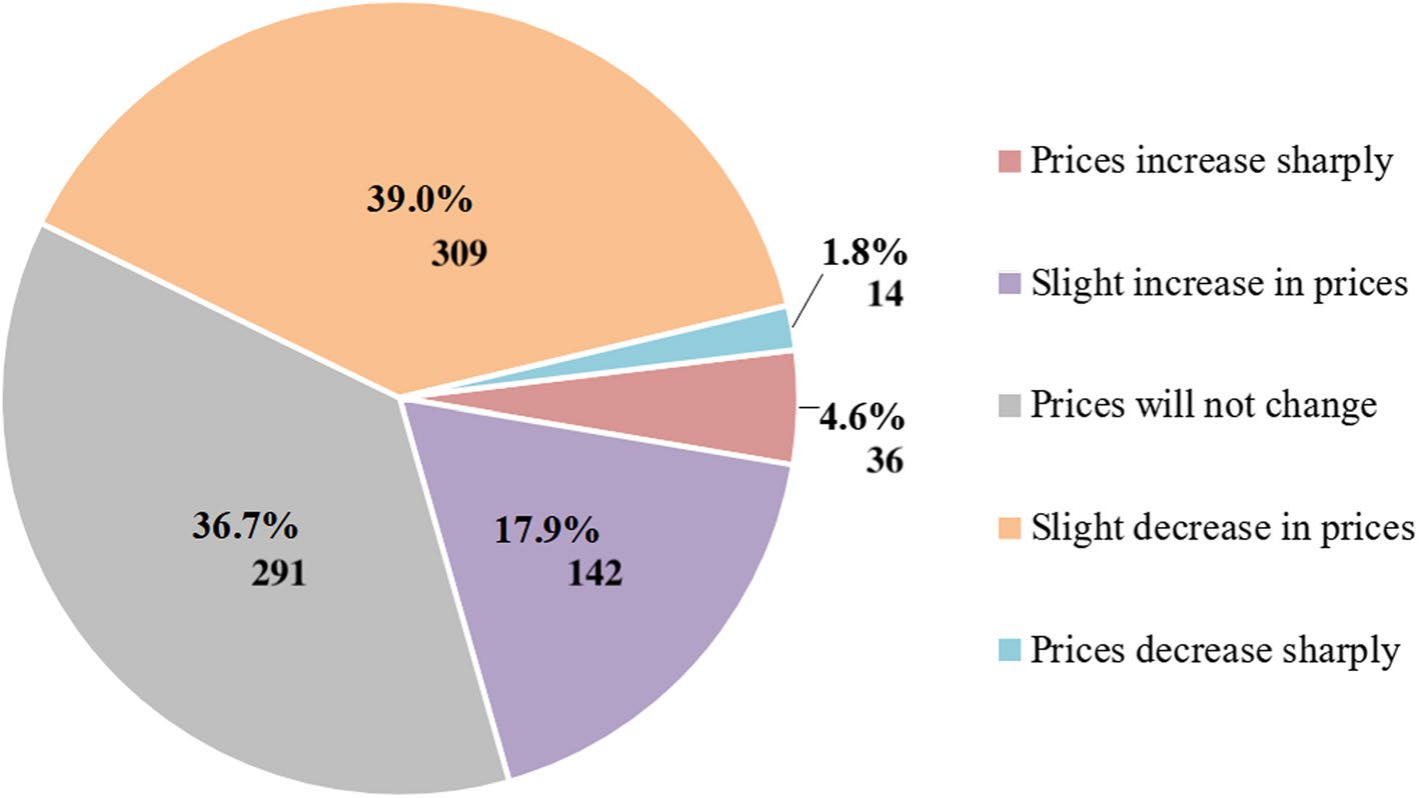
Distribution of consumer perceptions (%) of price changes following the legalisation of distance selling of medicines

This study reveals consumers’ perceptions of changes in medicine prices following the legalisation of remote retail sales of medicines (Fig. 3).

As can be seen from Fig. 3, the majority of consumers are convinced that the prices of medicines due to online sales will not change, will decrease slightly or even drop significantly. The smallest part of consumers assume that the distance selling of medicines will lead to a sharp increase in their prices.

One of the main aspects of the remote retail trade of medicines remains the delivery of medicines to the place of residence, stay or actual location of the buyer or to another address indicated by the buyer. As noted above, current Russian legislation does not contain any requirements for qualification of couriers who deliver medicines, or for their pharmaceutical or other specialized education.

The present study has identified who consumers think should deliver the medicines (Table 5).

**Table 5:** Distribution of consumer opinion (%) on who should deliver medicines to the address indicated by the customer

The medicine shall be delivered to the address specified by the purchaser	Opinions (%) consumers
Courier with a pharmaceutical education, only an employee of the pharmacy where the order is placed	11.9
Courier with medical or pharmacy education	22.0
Courier without medical or pharmaceutical education who cooperates with a pharmacy on the basis of an appropriate contract	26.1
Courier without medical or pharmaceutical education if there is a legal act making the courier responsible for the delivery of the medicines	30.7
I’m neutral about it	9.2

More than half of the consumers surveyed accept the possibility of medicine delivery by courier without pharmaceutical or medical education, but under certain conditions. As such conditions, consumers see the existence of a legal act making the courier responsible for the delivery of the requested medicines or the existence of an appropriate cooperation agreement between the courier and the pharmacy where the medicines are ordered. It should be noted that every third consumer admits the possibility of delivery by courier only if he/she has special education. Almost every fifth respondent believes that the courier must have a medical or pharmaceutical education. Even more stringent requirements for the courier are set by almost twice as few consumers, who are convinced that only pharmaceutical workers of the pharmacy where the order is placed should work with the medicines even during their delivery to the customers. A small proportion of consumers remain indifferent to the issue of delivery in distance retailing.

The present study assessed the strengths and weaknesses of the distance selling retail product according to consumers. Consumers were asked to select from two pre-formulated lists of the main strengths and weaknesses of distance selling of medicines, each of which had the most significance for consumers. The results, ranked in order of importance to consumers, are visualised in Tables 6 and 7.

**Table 6:** Distribution of consumer perceptions (%) of weaknesses in distance retailing

Weaknesses in remote retailing of pharmaceuticals	Opinions (%) of consumers
Existence of a risk of ineffective or unsafe medicine	71.6
Lack of confidence in the legitimacy of distance selling of medicines	48.2
Lack of pharmaceutical counselling in the choice of medicines	43.1
Increases the risk of medicine abuse and harm to health	31.2
Possible failure to deliver the medicine to the address specified by the purchaser	21.6
Difficulties in self-selection due to the availability of equivalents	19.7
Possibility of buying unnecessary medicines and wasting money	19.3
Lack of pharmaceutical counselling in the delivery of medicines	10.3
Possible overpricing of medicines	8.7
Inability to order some medicines (psychotropic medicines, etc.)	7.8
There are no weaknesses	7.6
The process of ordering and purchasing medicines is slowed down	7.3
Technical problems with the Internet	6.0

**Table 7:** Distribution of consumer opinions (%) on the strengths of remote retail sales of medicines

Strengths of remote retailing of medicines	Opinions (%) of consumers
Accessibility for persons with disabilities	80.3
Possibility to order a medicine at any time and place	77.5
Saves time (no time-consuming trip to the pharmacy)	76.6
Security during pandemic	74.5
Ability to self-identify the availability of necessary medicines	60.6
Ability to order different products in one place (on one website)	53.7
The ability to choose the right medicine from a wide range of products	40.4
Saving money (prices are lower than in a traditional pharmacy)	36.1
The ability to shop for medicines in the comfort of your home	31.7
Ability to obtain a large amount of information about the medicine	30.3
Lack of influence of the pharmacy worker on consumer choice	19.3
Absolute confidence in the efficacy and safety of the medicine	3.2
There are no strengths	3.2

The main disadvantages of distance retailing are perceived by consumers as the risk of receiving ineffective or unsafe medicines, lack of confidence that the pharmacy has a legal right (legal capacity) to sell medicines distantly, and lack of pharmaceutical counselling in selecting medicines. It should be noted that while the risk of receiving poor-quality medicines is, in the opinion of the vast majority of consumers, the most serious disadvantage of remote retail sales of medicines, the absolute confidence in the effectiveness and safety of medicines was mentioned by the smallest number of consumers as an advantage of this type of sales (Table 7).

A significant number of consumers also identified other disadvantages of distance retailing. These weaknesses include: the possibility of harm to health due to increased risk of medicine abuse; lack of delivery to the right address; difficulties in selecting medicines due to the numerous analogues; and the potential waste of money due to the purchase of unnecessary medicines. Lack of pharmaceutical counselling for medicine delivery is 4 times less of a concern for consumers than for medicine selection. The least significant disadvantage of the distance selling of pharmaceuticals, according to consumers, are problems in the functioning of the Internet on a technical level. At the same time, some consumers believe that this type of pharmaceutical trade has no weaknesses at all.

However, the weaknesses of online sales identified in this study can be compensated for by a number of perceived benefits of online sales (Table 7).

The greatest strengths of distance retailing were identified by an overwhelming majority of consumers (3/4 of respondents) as: accessibility for people with disabilities; the ability to order at any location and time; time savings; and safety during pandemic. Significant advantages of this type of trade for almost half of the respondents are the possibility to independently determine the availability of the required medicines, as well as to order different products at one site and choose the required medicine from a wide range of similar medicines. Almost every third consumer is convinced that remote retail sale of medicines saves money, allows to purchase medicines in comfortable conditions, and provides an opportunity to get a lot of information about the requested medicines. It should be noted that the absolute confidence in the efficacy and safety of medicines was mentioned by the smallest proportion of consumers as one of the advantages of Internet retailing.

## Discussion

In the current scientific literature, the topic of legalisation of remote retail trade in medicines has been the subject of research by leading Russian and foreign scientists.

Numerous studies show that the number of consumers who accept the option of buying medicines online increases over time. According to the present study, the number of consumers who fully support the purchase of OTC medicines online has increased by 1.5 times over time (31.7% in the first survey and 43.5% in the second survey), which is consistent with the results of USA researchers. Levaggi et al. [15] believe that the internet and e-commerce have revolutionised society, the economy and the world of health in general. But beyond the potential to improve consumer health, the Internet can also pose major risks to consumer health. A study by the authors found that the strategies used by online pharmacies vary according to the quality, quantity, and target group of the medicines in question, mainly targeting groups of consumers who wish to use the medicines without consulting a doctor.

However, the Internet is a limitless and low-controlled space. Almost half of the respondents (48.2%) do not trust Internet sales of medicines due to a lack of confidence in the legitimacy of distance selling from the pharmacy where they purchase the product. This correlates with the results obtained by Hungarian scientists (András Fittler, Róbert György Vida, Mátyás Káplár and others). The scientists [16] point out that over the past two decades the Internet has become the accepted way to purchase products and services, and buying medicines online is no exception. Although thousands of Internet pharmacies are available online, the real size of the market is unknown. Consumers who use the Internet more and buy products online are more likely to buy medicines remotely. However, there is a risk of buying medicines online from illegitimate sites. Illegitimate websites that sell fake or substandard medicines and pose as online pharmacies pose a serious risk in the distance selling of medicines. Such unregistered pharmacies may exploit consumers’ trust and cause harm to their health.

Nevertheless, despite the uncertainty about the legitimacy of pharmacies selling medicines online, the number of respondents who fully support the online sale of prescription medicines doubled during the study period (9.2% in the first and 19.6% in the second survey, respectively). At the same time, a study authored by Grazia Orizio, Peter Schulz, Serena Domenighini and other scientists from England proved that only 51 pharmacies (out of 118 surveyed) (43.2%) indicated the exact location. 81.4% of online pharmacies did not require a prescription from the physician. This suggests that the issue of regulating the websites of online pharmacies and the online sale of prescription medicines requires additional adjustment [17].

Similar observations have been documented by other studies. For example, a study by British scientists Boyd et al. [18] found that online pharmacies that confirmed their UK location (25%) required a prescription before purchasing antibiotics and were appropriately registered. Online pharmacies without a location (50%) had various prescription requirements, but were not properly registered. 45% of online pharmacies did not require a prescription before purchase. In 80% of the Internet pharmacies, the consumer decided on the choice, dosage and quantity of antibiotics. The authors’ study highlights safety concerns for customers purchasing antibiotics online. The authors conclude that regulations are urgently needed for online antibiotic suppliers.

Therefore, online pharmacies that are not authorised to dispense medicines online should be strictly controlled.

Mackey and Nayyar from England [19] also state that there is an urgent need for finding international consensus, more research and technology development to combat illegal online pharmacies.

Thanks to many studies aimed at identifying the cause of widespread illegal Internet pharmacies, such as the scientific work of Vida et al. [20] or Penley et al. [21], the global pharmaceutical community has concluded that online surveillance bodies need to be improved.

Studies by Abanmy [22] and Fittler et al. [23] have highlighted the need for concerted and targeted action by international organizations to tackle uncontrolled sales of counterfeit and substandard medicines.

At the same time, it is necessary to carry out preventive work with consumers.

For example, Italian researchers, Orizio et al. [24], believe that a two-tiered approach should be adopted to increase the benefits and minimise the risks associated with online pharmacies. The first level should focus on policy - laws should regulate Internet pharmacies internationally. The second level should focus on the individual. This approach is to increase the health literacy needed to make good health-related choices, recognise the risks and maximise many opportunities offered by the world of medicine.

Many studies aim to determine the level of consumer awareness of the risks of buying medicines online and how to protect themselves from buying a substandard product. The present study also addresses the above issue. Our findings show that consumers perceive online sales of medicines to have weaknesses: the risk of receiving ineffective or unsafe medicines (71.6%), lack of confidence in the eligibility of distance selling medicines (48.2%), lack of pharmaceutical counselling in choosing medicines (43.1%), risk of harmful health consequences (31.2%) and others. However, selling drugs online has undeniable strengths. The advantages of remote retail sales were recognised by consumers. Most consumers of this study include accessibility for persons with disabilities (80.3%), the ability to order drugs at any place and time (77.5%), saving time (76.6%) and safety in a pandemic (74.5%). Meanwhile, in Saudi Arabia, the purchase of medicines online has become increasingly relevant over the past three decades among the consumers. The scientists Alwhaibi et al. [25], emphasise that consumer perception and understanding of how safe or risky it is to purchase medicines online is very important. Overall, 36% of participants in the authors’ study had ever purchased a product online. The most common positive motivating factors were lower costs (55.7%), easy access to the Internet (54.1%), a wide range of medicines (52.6%) and confidentiality (43.6%). About 60.4% of participants thought that it could be safe to buy medicines online. Only 32.7% of participants distinguished between registered and unlicensed commercial websites. The results of the authors’ study clearly demonstrate the need in Saudi Arabia to raise awareness of the safety of obtaining medicines remotely.

One of the main benefits of distance retailing is the minimum of social contact when buying a medicine, which was a lifesaver when the COVID-19 pandemic struck the world in 2020, challenging mankind. In the Russian Federation, for the first time, remote retail sale of medicines for medical use was legalized, which are dispensed from pharmacies without a doctor’s prescription. And also, as an exception, rules have been adopted that stipulate the possibility of selling prescription medicines in emergency situations, such as a pandemic, for example. During this challenging time, pharmaceutical workers have had to change their strategies for dealing with public health emergencies. Nadeem et al. [26], as well as Hasen et al. [27] came to similar results after conducting research in their countries.

According to researchers from China such as, Lia et al. [28], or for example, Liu et al. [29], pharmacists play a vital role in guiding the pharmaceutical industry and faced with the COVID-19 pandemic, pharmacists were able to fully utilize their professional expertise, rationally analyse the current situation, rapidly formulate telemedicine strategies, and work together and effectively deliver innovative pharmaceutical services to ensure the safety and rational use of medicines.

Scientists from Italy: Baldoni et al. [30] – or from Spain: Tortajada-Goitia et al. [31] – believe that the pandemic has become the starting point for the development of many innovative methods of pharmaceutical care, including telepharmacy (a form of pharmaceutical care in which pharmacists and patients are located in different locations and can interact using information technologies).

One of the main issues in distance retailing of medicines is their delivery. Several countries have opted for postal services as the safest way to deliver goods to customers.

The authors of scientific papers on the delivery of mail-order medicines ordered online are Fernandez et al. [32], as well as Neil et al. [33] believe that postal orders offer patients a better chance of achieving good adherence to medicines with the right dosages and amounts taken.

However, a study by Schmittdiel et al. [34] from the United States uncovered the barriers and factors faced by mail-order customers. Barriers to the use of mail-order pharmacies included: unpredictability of medicine delivery dates, concerns about mail safety and the difficulty of coordinating refill orders across multiple prescriptions. However, mail-order pharmacy delivery offers greater access and convenience (e.g., no need to queue). Nowadays, in the Russian Federation, Russian Post is now known to deliver medicines ordered online. In partnership with industry aggregator platforms and marketplaces, Russian Post [35] is responsible for logistics and product operations, and platform solutions ensure information exchange between pharmacies, distributors, pharmaceutical companies and Russian Post warehouses.

## Conclusion

Remote retailing of medicines is actively developing in many countries around the world and is gaining more and more followers every day and is an innovative and relevant trend in the Russian pharmaceutical market. This study shows that different age groups of consumers are showing a positive trend towards distance retailing of medicines. There is an increase in the number of consumers who fully support the legalisation of the sale of medicines that are dispensed from pharmacies strictly in accordance with a doctor’s prescription. There has been a decrease in the number of respondents who need pharmacy counselling when choosing medicines online. Thus, consumer loyalty to distance retailing of medicines is increasing and the demand for online purchasing is growing. Accordingly, in the near future, the number of medicines sold online and the number of pharmacies providing remote purchase services will increase. However, global experience suggests that the sale of medicines online should continue to be tightly regulated.

### The strength of the study

The study was conducted in the central region of the Russian Federation and covered a sufficient sample size to ensure representativeness of the data. The present study can form the basis for further research in finding the most effective and safest way to provide pharmaceutical services remotely. The data obtained in this study may be useful for planning further productive activities of pharmacies that retail medicines online, as well as for the authorities that regulate the remote sale of medicines.

### Limitations of the study

The study has some limitations, which are related to the limited mobility of consumers during the COVID-19 pandemic and a limited time available to respondents for answering the researcher’s questions. In order to facilitate data collection, the study was conducted using a specially designed structured questionnaire, most of the questions being formulated in closed form (with a pre-determined choice of answers) or semi-closed form (pre-determined choice of answers with an option for respondents to answer independently).

## Data Availability

The datasets used and/or analysed during the current study available from the corresponding author on reasonable request.
